# Vaccination as a Promising Approach in Cardiovascular Risk Mitigation: Are We Ready to Embrace a Vaccine Strategy?

**DOI:** 10.3390/biom14121637

**Published:** 2024-12-20

**Authors:** Georgios Tsioulos, Natalia G. Vallianou, Alexandros Skourtis, Maria Dalamaga, Evangelia Kotsi, Sofia Kargioti, Nikolaos Adamidis, Irene Karampela, Iordanis Mourouzis, Dimitris Kounatidis

**Affiliations:** 1 Fourth Department of Internal Medicine, Medical School, Attikon General University Hospital, National and Kapodistrian University of Athens, 12462 Athens, Greece; geotsioulos@med.uoa.gr; 2 First Department of Internal Medicine, Sismanogleio General Hospital, 15126 Athens, Greece; skargioti@gmail.com (S.K.); nikos.adamidis7@gmail.com (N.A.); 3 Department of Internal Medicine, Evangelismos General Hospital, 10676 Athens, Greece; alex.skourtis@gmail.com; 4 Department of Biological Chemistry, National and Kapodistrian University of Athens, 75 Mikras Asias Str., 11527 Athens, Greece; madalamaga@med.uoa.gr; 5 Second Department of Internal Medicine, Medical School, National and Kapodistrian University of Athens, Hippokratio General Hospital, 11527 Athens, Greece; lila.kotsi@yahoo.com; 6 Second Department of Critical Care, Medical School, Attikon General University Hospital, University of Athens, 12461 Athens, Greece; eikaras1@gmail.com; 7 Department of Pharmacology, National and Kapodistrian University of Athens, 11527 Athens, Greece; imour@med.uoa.gr; 8 Diabetes Center, First Department of Propaedeutic Internal Medicine, Medical School, National and Kapodistrian University of Athens, Laiko General Hospital, 11527 Athens, Greece; dimitriskounatidis82@outlook.com

**Keywords:** apolipoprotein B100, atherosclerosis, autoantigens, cardiovascular disease, cholesteryl ester transfer protein, immunization, low-density lipoprotein cholesterol, proprotein convertase subtilisin/kexin type 9, vaccination

## Abstract

Cardiovascular disease (CVD) remains a leading global health concern, with atherosclerosis being its principal cause. Standard CVD treatments primarily focus on mitigating cardiovascular (CV) risk factors through lifestyle changes and cholesterol-lowering therapies. As atherosclerosis is marked by chronic arterial inflammation, the innate and adaptive immune systems play vital roles in its progression, either exacerbating or alleviating disease development. This intricate interplay positions the immune system as a compelling therapeutic target. Consequently, immunomodulatory strategies have gained increasing attention, though none have yet reached widespread clinical adoption. Safety concerns, particularly the suppression of host immune defenses, remain a significant barrier to the clinical application of anti-inflammatory therapies. Recent decades have revealed the significant role of adaptive immune responses to plaque-associated autoantigens in atherogenesis, opening new perspectives for targeted immunological interventions. Preclinical models indicate that vaccines targeting specific atherosclerosis-related autoantigens can slow disease progression while preserving systemic immune function. In this context, numerous experimental studies have advanced the understanding of vaccine development by exploring diverse targeting pathways. Key strategies include passive immunization using naturally occurring immunoglobulin G (IgG) antibodies and active immunization targeting low-density lipoprotein cholesterol (LDL-C) and apolipoproteins, such as apolipoprotein B100 (ApoB100) and apolipoprotein CIII (ApoCIII). Other approaches involve vaccine formulations aimed at proteins that regulate lipoprotein metabolism, including proprotein convertase subtilisin/kexin type 9 (PCSK9), cholesteryl ester transfer protein (CETP), and angiopoietin-like protein 3 (ANGPTL3). Furthermore, the literature highlights the potential for developing non-lipid-related vaccines, with key targets including heat shock proteins (HSPs), interleukins (ILs), angiotensin III (Ang III), and a disintegrin and metalloproteinase with thrombospondin motifs 7 (ADAMTS-7). However, translating these promising findings into safe and effective clinical therapies presents substantial challenges. This review provides a critical evaluation of current anti-atherosclerotic vaccination strategies, examines their proposed mechanisms of action, and discusses key challenges that need to be overcome to enable clinical translation.

## 1. Introduction

Cardiovascular disease (CVD) remains the leading cause of mortality worldwide, responsible for approximately 18 million deaths annually and accounting for 32% of all global deaths. CVD encompasses a range of conditions, including coronary heart disease (CHD), cerebrovascular disease (CeVD), peripheral arterial disease (PAD), and other cardiovascular disorders. Notably, over 80% of CVD deaths result from heart attacks and strokes, with one-third of these deaths occurring prematurely in individuals under the age of 70 [1]. The primary pathological process underlying CVD is atherosclerosis, a chronic and progressive arterial condition marked by successive stages, which are endothelial dysfunction, fatty streak formation, fibrous plaque development, and ultimately plaque rupture. Atherosclerosis is influenced by various cardiovascular (CV) risk factors, but both experimental and clinical evidence emphasize the pivotal role of inflammation in its progression and associated complications [2]. Notably, emerging research highlights chronic low-grade inflammation as a unifying link among traditional atherosclerosis risk factors. This interplay reflects a continuous interaction between metabolic and immune pathways, involving activation of the innate immune system and the dysregulation of adaptive immunity [3].

Atherosclerosis involves a complex interplay of pro-inflammatory and anti-inflammatory pathways, resulting in a diverse immune cell environment within atherosclerotic plaques [4]. The process of atherogenesis is initiated by endothelial dysfunction in large- and medium-sized arteries, especially in regions subject to turbulent blood flow and low shear stress. Hemodynamic factors, together with traditional CV risk factors such as hypercholesterolemia, hypertension, smoking, obesity, and diabetes mellitus, are influenced by genetic, dietary, lifestyle, and environmental factors. These elements disrupt the balance between atheroprotective anti-inflammatory responses and proatherogenic inflammatory responses within the vascular endothelium, ultimately favoring the latter [5]. This imbalance leads to the buildup of cholesterol-rich apolipoprotein B (ApoB)-containing lipoproteins, primarily low-density lipoprotein cholesterol (LDL-C) particles, in the arterial intima. This accumulation recruits inflammatory cells, particularly macrophages and T cells, and triggers the release of pro-inflammatory cytokines, contributing to the formation of atherosclerotic plaques [6]. Advanced plaques may erode or rupture, resulting in arterial thrombotic events that can lead to major adverse cardiovascular events (MACEs) [7].

Current clinical practices for preventing and treating atherosclerosis focus on lifestyle modifications and therapeutic strategies that target key risk factors, especially hypercholesterolemia. Most therapies target the reduction in low-density lipoprotein cholesterol (LDL-C) levels, primarily through the use of statins, which inhibit the rate-limiting enzyme in cholesterol biosynthesis. In recent years, proprotein convertase subtilisin/kexin type 9 (PCSK9) inhibitors have revolutionized the treatment landscape, providing substantial benefits in selected cases. These agents function by increasing LDL-C receptor levels on hepatocytes, thereby enhancing the clearance of LDL-C from the circulation [8,9]. Consequently, targeting the inflammatory component of atherosclerosis presents a promising approach to addressing this unmet need for residual CV risk reduction.

Pioneering studies, such as the CANTOS (Canakinumab Anti-inflammatory Thrombosis Outcome Study) and COLCOT (Colchicine Cardiovascular Outcomes Trial) [10,11], have highlighted the potential benefits of anti-inflammatory interventions in reducing MACEs [12]. However, both canakinumab and colchicine have been associated with an increased incidence of adverse events, particularly infections, which led to higher mortality rates [10,11]. These findings underscore the urgent need for more targeted immunomodulatory strategies in the management of atherosclerosis, approaches that can enhance efficacy, durability, and safety [12]. Building on the remarkable success of active immunization against various infectious diseases, research has developed vaccines for atherosclerosis that target specific components of the cholesterol homeostasis pathway or key elements of the immune network involved in the disease [13].

This review aims to dissect the cellular and molecular mechanisms underpinning atherosclerosis while critically evaluating the current landscape of immunomodulatory therapies, with an emphasis on vaccination strategies. Additionally, it seeks to address fundamental questions about human immune responses to key atherosclerotic epitopes and the development of vaccine technologies. Given the considerable challenges that remain, this review highlights the barriers that must be overcome to successfully translate anti-atherosclerotic vaccines into clinical practice.

## 2. Inflammation and Immune Dynamics in Atherosclerosis

### 2.1. Foundations of Atherogenesis: The Role of Inflammation and Immune Activation

Extensive research has highlighted inflammation as a core driver in atherosclerosis, specifically detailing how it triggers both innate and adaptive immune responses, weaving these interactions into a complex network. Innate immune cells, including monocytes, macrophages, neutrophils, mast cells, and dendritic cells (DCs), play pivotal roles by producing cytokines and chemokines, activating phagocytosis and complement pathways, and presenting antigens to initiate adaptive immune responses [14]. The adaptive immune response, which follows the onset of atherosclerosis, is closely linked to antigen presentation by antigen-presenting cells (APCs) and a cytokine-rich milieu generated by innate immune cells. Key players in adaptive immunity include CD4^+^ and CD8^+^ T cells, B cells, and innate lymphoid cells (ILCs), with a unique subset called natural killer (NK) T cells and non-cytotoxic ILC groups 1, 2, and 3, bridging innate and adaptive immunity [15].

Atherosclerosis begins with endothelial dysfunction, enabling LDL-C particles to infiltrate the subendothelial space of large and medium arteries. Once trapped by proteoglycans and other extracellular matrix elements, LDL-C particles undergo further modification through enzymatic actions, including those of matrix lipases and proteases, which enhance their binding affinity. These retained LDL-Cs are then oxidized by intimal free radicals, such as superoxide (O_2_•^−^) and hydroxyl radicals (•OH), as well as by enzymes like myeloperoxidases and lipoxygenases. Oxidized low-density lipoprotein cholesterol (OxLDL-C) serves as a pro-inflammatory agent, triggering endothelial cells to express adhesion molecules, such as vascular cell adhesion molecule-1 (VCAM-1), intercellular adhesion molecule-1 (ICAM-1), and e-selectin, along with chemokines like C-X3-C motif chemokine ligand 1 (CX3CL1) and C-C motif chemokine ligand 2 (CCL2), which recruit circulating monocytes into the arterial intima. Neutrophils are also attracted to the inflamed intima via chemokines, including CXCL1, CXCL2, and CXCL8, where they release granule proteins that amplify monocyte adhesion and migration [16]. Endothelial cells and other local cells further contribute by releasing cytokines like macrophage colony-stimulating factor (M-CSF) and granulocyte–macrophage colony-stimulating factor (GM-CSF), driving the differentiation of monocytes into macrophages within the arterial intima [17].

### 2.2. Foam Cell Formation and Atherosclerotic Plaque Development

Foam cell formation, driven by macrophages and vascular smooth muscle cells (VSMCs), is central to atherogenesis. Macrophages internalize OxLDL-C via scavenger receptors, while VSMCs migrate into the intima, transdifferentiate into macrophage-like cells, and also form foam cells. These VSMC-derived foam cells constitute approximately 50% of the foam cell population in human coronary intima [18,19,20]. Early immune cell accumulation (e.g., DCs, macrophages, T cells) and lipid-laden foam cells form fatty streaks, the precursors to atherosclerotic lesions [21]. OxLDL-C and cholesterol crystal formation perpetuate inflammation by activating nuclear factor-κB (NF-κB) and the NOD-like receptor protein 3 (NLRP3) inflammasome, leading to interleukin (IL)-1β and IL-18 release. Foam cells exhibit some anti-inflammatory gene expression, but oxidative stress favors pro-inflammatory and pro-atherogenic responses [22,23,24,25]. Foam cell apoptosis, initially cleared by efferocytosis, increases as the disease progresses. Dysregulated efferocytosis and oxidative stress lead to secondary necrosis, releasing pro-inflammatory mediators like cholesterol crystals, further destabilizing plaques by activating complement, metalloproteinases, and tissue factor (TF). This contributes to necrotic core formation, a hypocellular lipid-rich area encapsulated by a fibrous cap composed of VSMCs and the extracellular matrix. The necrotic core and fibrous cap signify advanced atherosclerosis [7,26,27].

## 3. Adaptive Immunity and Cellular Interactions in Atherosclerosis

### 3.1. T Cells and Antigen Presentation in Atherogenesis

In atherogenesis, adaptive immune responses are primarily mediated by T lymphocytes and APCs. Single-cell RNA sequencing and mass cytometry confirm the presence of aortic T cells across all stages of atherosclerosis in mice, as well as in the adventitia of healthy arteries in both wild-type and genetically predisposed mouse models [28,29,30]. Chemotactic factors, including CCL5 and CXCL16, recruit circulating T cells to atherosclerotic lesions [31,32]. Early arterial walls predominantly host regulatory T (Treg) cells, but this shifts to CD4^+^ effector T cells (T helper cells) during atherosclerosis onset, with Treg reduction impairing immune tolerance to self-antigens and facilitating disease progression [33]. Natural Tregs (nTregs), thymus-derived and forkhead box protein P3 (FOXP3)-expressing, maintain immune tolerance and balance. Inducible Tregs (iTregs), derived from peripheral nTregs, exhibit phenotypic plasticity but may lose FOXP3 expression and transition into ex-Treg cells. Research links these unstable Treg subsets to atherogenesis, while certain Tregs have demonstrated atheroprotective roles in human studies [34,35]. Arterial APCs present atherosclerosis-associated peptides to naïve CD4^+^ and CD8^+^ T cells in secondary lymphoid organs via major histocompatibility complex (MHC) molecules, alongside co-stimulatory signals and cytokines that drive T cell polarization. Polarized T cells, including Th cell subsets and cytotoxic T lymphocytes (CTLs), re-enter circulation and accumulate in plaques. Within plaques, Treg cells suppress adaptive immunity through IL-10, transforming growth factor-beta (TGF-β) and contact-dependent mechanisms, promoting tolerogenic APC phenotypes in dendritic cells and macrophages [4,14].

### 3.2. Th Cell Polarization, Cytotoxic T Cells, and B Cells in Plaque Development

Th cells and CTLs play pivotal roles in plaque development as intimal APCs trigger recall responses via cytokines and co-stimulatory signals. Th1 cells, the predominant Th subset in plaques, exert pro-inflammatory effects by producing interferon-gamma (IFN-γ) and tumor necrosis factor-alpha (TNF-α), which enhance endothelial adhesion molecules, upregulate CCR5, and drive foam cell formation. Th2 cells, producing IL-4, IL-5, and IL-13, exhibit mixed roles—recent evidence suggests they may reduce monocyte recruitment, promote anti-inflammatory M2 macrophages, and enhance collagen deposition for atheroprotection, but some studies link them to pro-atherogenic effects as well. Th17 cells, characterized by IL-17A secretion, demonstrate context-dependent plasticity, contributing both pro- and anti-atherogenic effects [15,36,37].

CD8^+^ CTLs are predominantly pro-atherogenic, releasing TNF-α and IFN-γ to induce Fas receptor-mediated apoptosis and cell lysis via granzymes and perforin, as well as to amplify inflammation through MHC-I upregulation. While their abundance in advanced lesions underscores their pathogenic role, certain CD8^+^ subsets may exhibit neutral or protective effects [38,39]. B cells contribute through both adaptive and innate responses, producing antibodies and cytokines and acting as APCs. B1 cells, secreting natural IgM antibodies against OxLDL-Cs, protect against foam cell formation and exhibit atheroprotective properties. Conversely, B2 cells, activated by T follicular helper (Tfh) cells, produce immunoglobulin G (IgG) antibodies after class-switching, which are associated with atherosclerosis progression. Tertiary lymphoid organs, composed of Tfh cells, B cells, and plasma cells, form in advanced plaques, facilitating localized antibody production and promoting lesion development [38,40,41].

**Figure 1:**
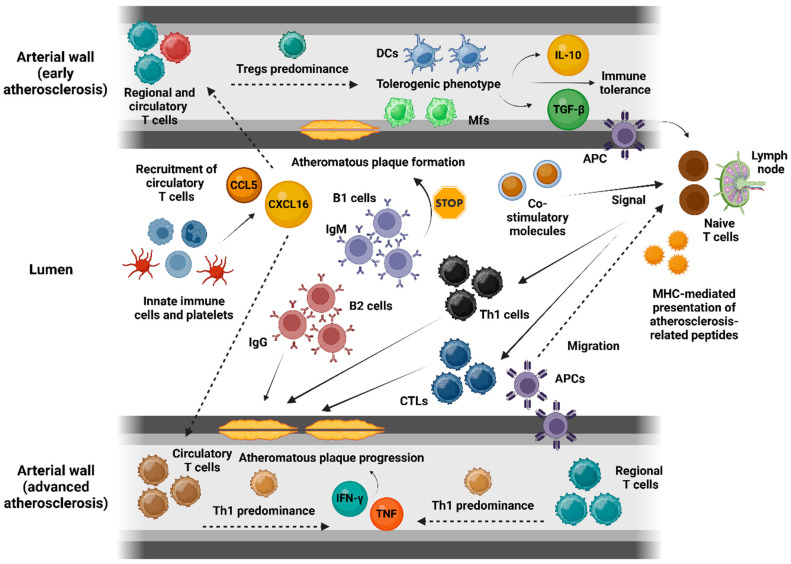
Schematic representation of adaptive immune cell involvement in the progression of atherosclerosis. In early atherosclerosis stages, Tregs accumulate in arterial walls, interacting with APCs like dendritic cells and macrophages, releasing anti-inflammatory cytokines (IL-10, TGF-β) to maintain immune tolerance and reduce inflammation. Circulating T cells also migrate to plaques, attracted by chemotactic signals (CCL5, CXCL16) from platelets and immune cells. As the disease progresses, APCs present atherosclerosis-related antigens to naïve T cells in regional lymph nodes, driving T cell polarization into pro-inflammatory Th cells and CTLs. These activated T cells return to plaques, exacerbating local inflammation. In advanced stages, Th1 cells dominate, secreting IFN-γ and TNF-α to promote plaque growth and instability. B cells modulate disease progression: B1 cells secrete protective IgM antibodies, while B2 cells release IgG antibodies, contributing to atherosclerosis progression. Overall, the adaptive immune response shifts from protective to pathogenic, driving plaque progression and vascular damage [14,15,16,17,18,19,20,21,22,23,24,25,26,27,28,29,30,31,32,33,34,35,36,37,38,39,40,41]. Abbreviations: APCs: antigen-presenting cells; B1 cells: B cell subtype 1; B2 cells: B cell subtype 2; CCL5: chemokine (C-C motif) ligand 5; CTLs: cytotoxic T lymphocytes; CXCL16: chemokine (C-X-C motif) ligand 16; DCs: dendritic cells; IFN-γ: interferon gamma; IgG: immunoglobulin G; IgM: immunoglobulin M; IL-10: interleukin-10; Mfs: macrophages; MHC: major histocompatibility complex; T cells: T lymphocytes; TGF-β: transforming growth factor beta; Th cells: T helper cells; TNF: tumor necrosis factor; Tregs: regulatory T cells. Created in BioRender. Kounatidis, D. (2024) https://BioRender.com/x00b831, assessed on 24 November 2024.

Table 1 summarizes the key areas of the pathophysiology of atherosclerosis, focusing on the roles of inflammation and immune activation, atherosclerotic plaque development, and the involvement of T cells, antigen presentation, and various immune cells in the progression of the disease. Additionally, Figure 1 illustrates the dynamic role of adaptive immune cells in atherosclerosis progression, depicting how early regulatory and protective responses shift toward pro-inflammatory actions that drive plaque growth and instability as the disease advances.

## 4. Immunomodulatory Strategies Toward Atherosclerosis Vaccine Development

The remarkable advancements in understanding the immunopathogenesis of atherosclerosis have fueled the development of promising innovative therapeutics aimed at modulating innate and adaptive immunity to counteract atherosclerotic progression. These include novel immunomodulatory strategies as well as established anti-inflammatory agents with potential immunological benefits [42]. Current approaches encompass colchicine, monoclonal antibodies targeting pro-inflammatory cytokines like IL-1β and IL-6, low-dose IL-2, CD8^+^ T cell and B2 cell depletion therapies, and passive and active immunization targeting specific atherosclerosis-associated antigens [42].

### 4.1. Colchicine and Anti-Inflammatory Agents

Colchicine, a long-known anti-inflammatory drug, inhibits myeloid cell trafficking via the suppression of actin polymerization [43]. Its potential as an atheroprotective agent has been validated in pivotal clinical trials such as COLCOT and LoDoCo2 (Low Dose Colchicine 2), both of which demonstrated a significant reduction in MACEs [11,44]. These findings led to the U.S. Food and Drug Administration’s (FDA) approval of colchicine for anti-atherosclerotic indications [45,46]. Despite these advances, colchicine’s exact atheroprotective mechanism and its contraindications in severe chronic kidney disease (CKD) (estimated glomerular filtration rate < 30 mL/min) remain unresolved, limiting its broader clinical application. Conversely, other conventional anti-inflammatory agents, including glucocorticoids, nonsteroidal anti-inflammatory drugs (NSAIDs), and janus kinase (JNK) inhibitors, have been associated with increased CV risks, including thrombotic events and complications following acute coronary syndromes [47,48,49]. Methotrexate, despite its efficacy in rheumatoid arthritis and connective tissue diseases, failed to demonstrate cardiovascular protection in stable atherosclerosis patients in a large-scale trial [50].

### 4.2. Cytokine-Related Therapies

The CANTOS trial pioneered the investigation of IL-1β inhibition using canakinumab, demonstrating reduced MACEs in myocardial infarction (MI) survivors with residual inflammation. However, an increased risk of infection raised significant safety concerns [10]. The study’s findings spurred interest in targeting downstream cytokines such as IL-6. Ziltivekimab, an IL-6 ligand-inhibiting antibody, showed promising reductions in pro-inflammatory and prothrombotic biomarkers in CKD patients with residual inflammation during the RESCUE (IL-6 inhibition with ziltivekimab in patients at high atherosclerotic risk) trial [51]. These encouraging phase II results have led to an ongoing phase III trial to evaluate its broader CV impact (NCT05021835).

Tregs are central to maintaining immune homeostasis by suppressing pro-atherogenic effector T cell responses. The observation that Tregs expand in response to ultra-low doses of IL-2 prompted the exploration of low-dose IL-2 therapy for CVD. The LILACS (Low-Dose Interleukin-2 in Patients With Stable Ischemic Heart Disease and Acute Coronary Syndromes) study demonstrated significant Treg expansion without adverse events in patients with stable or unstable ischemic heart disease [52]. Building on these findings, the phase II IVORY trial is investigating the impact of low-dose IL-2 on vascular inflammation, with results pending (NCT04241601).

### 4.3. CD8^+^ T Cell and B2 Cell Modulation

CD8^+^ T cells, traditionally considered pro-atherogenic, have been implicated in both atherosclerosis progression and its complications [53,54]. The experimental depletion of CD8^+^ T cells in murine models ameliorated atherosclerotic development, supporting the potential of CD8^+^-targeting monoclonal antibodies as therapeutic agents. Advances in chimeric antigen receptor (CAR) T cell technology further expand the horizon for immune-based therapies, with preliminary studies showing efficacy in preventing cardiac fibrosis via CAR T cells targeting cardiac fibroblasts [55].

In contrast, B2 cells have a complex role in atherosclerosis. While their production of proatherogenic IgG antibodies is well-documented, they also contribute to disease progression through cytokine release, dendritic cell activation, and T cell co-stimulation [56]. Preclinical research in mice demonstrated that B2 depletion using anti-CD20 antibodies reduced atherosclerosis and MI-associated complications without affecting protective IgM-producing B1 cells [57]. The safety of the anti-CD20 monoclonal antibody rituximab has been confirmed in MI patients, with ongoing investigations exploring its effects on cardiac remodeling post-MI (NCT05211401).

Table 2 summarizes some of the most prominent preclinical and clinical studies concerning immunomodulating agents against atherosclerosis.

### 4.4. Vaccination-Based Approaches

Vaccination strategies represent a novel avenue for atherosclerosis management. Passive immunization with naturally occurring anti-oxLDL-C IgG antibodies offers a simple mechanism, while active immunization with lipid or non-lipid antigens targets atherogenesis through robust humoral responses. Some vaccines, such as those targeting oxLDL-C and ApoB100, have demonstrated additional benefits by mitigating existing pro-atherogenic immune responses, achieving atheroprotection through immune tolerance induction [13].

These innovative approaches exemplify the growing potential of immunomodulation in addressing the inflammatory underpinnings of atherosclerosis. Future research must further elucidate the mechanisms and optimize the clinical applicability of these therapies.

## 5. Passive Immunization Using Naturally Occurring IgG Antibodies

Numerous studies have established a link between atherosclerotic disease and serum antibodies targeting LDL-C and ApoB in untreated mice and humans. These studies have observed an increase in antibody titers following vaccination with LDL-C or ApoB antigens [58]. Notably, while most naturally occurring IgM antibodies against oxidation-specific epitopes exhibit pronounced atheroprotective effects, the precise physiological role of IgG antibodies remains unclear [59]. Preclinical data further highlight the ambiguous role of anti-oxLDL-C IgG antibodies in animal models, as these antibodies have demonstrated both pro- and anti-atherogenic responses in the context of passive vaccination [60,61].

In humans, immunotherapy using injectable immunoglobulins was initially evaluated in the GLACIER trial, a randomized, double-blind, phase II study designed to assess the efficacy of an injectable anti-oxLDL-C monoclonal antibody in patients with stable carotid or aortic plaque lesions [62]. This trial investigated a human recombinant IgG1 antibody (MLDL1278A) targeting the oxidation-specific epitope p45 of ApoB. The antibody was hypothesized to attenuate atherosclerosis-related inflammation by forming immune complexes with oxidized LDL-Cs, thereby preventing the activation of inflammatory macrophages within plaques. However, despite its demonstrated efficacy in murine and primate models [63,64], MLDL1278A failed to significantly reduce arterial inflammation in human subjects, as assessed by serial fluorodeoxyglucose–positron emission tomography (FDG-PET) imaging [65]. This lack of efficacy was attributed to several methodological limitations, including a small study population and a short follow-up period, which complicated the interpretation of results [62,65].

Natural IgM antibodies against phosphorylcholine (PC) have long been recognized for their protective effects against atherosclerosis and CVD, a finding corroborated by recent population-based studies, such as a Swedish cohort study involving women [66,67]. Preclinical studies investigating IgG anti-PC antibodies have shown promising results, suggesting a reduction in vascular inflammation potentially mediated by a decreased macrophage uptake of ox-LDL-C [68]. In 2021, a phase IIa, placebo-controlled, double-blind study assessed the safety and efficacy of a single intravenous dose of ATH3G10, a fully human IgG1 antibody against phosphorylcholine, in high-risk patients with ST-segment elevation myocardial infarction (STEMI). The trial aimed to evaluate its impact on left ventricular remodeling and recurrent cardiovascular events. However, the results of this study remain unpublished at the time of writing [NCT03991143].

More recently, a placebo-controlled phase IIa trial assessed orticumab, a fully human IgG1 antibody against malondialdehyde (MDA)-ApoB100, for its ability to reduce coronary plaque burden in high-risk psoriasis patients [69]. Consistent with earlier animal studies [60], orticumab demonstrated a reduction in coronary inflammation, as evaluated through imaging biomarkers.

## 6. Active Immunization with Lipid-Related Antigens

### 6.1. Vaccination Strategies Targeting Lipoproteins and Apolipoproteins

#### 6.1.1. Low-Density Lipoprotein Cholesterol (LDL-C)

LDL-C particles consist of a lipid core primarily composed of cholesteryl ester molecules, with smaller amounts of triglycerides (TGs) and unesterified cholesterol. This core is surrounded by a phospholipid monolayer containing a single molecule of ApoB100 [70]. The infiltration of LDL-C particles into the arterial intima is a fundamental event in the pathogenesis of atherosclerosis. Within the intima, LDL-C particles undergo oxidative modifications, forming oxLDL-Cs, which are highly immunogenic. Unlike native LDL-C, oxLDL-C activates innate and adaptive immune responses, synergistically driving atherogenesis [71].

The phagocytosis of oxLDL-C by macrophages is pivotal in atherogenesis and leads to two key outcomes. First, macrophages transform into foam cells, which are proatherogenic and amplify inflammation, thereby contributing to the enlargement of the lipid core. Second, macrophages function as APCs, displaying oxLDL-C-derived epitopes to CD8^+^ and CD4^+^ T cells via MHC-I and MHC-II molecules. This process generates effector T cells, predominantly proatherogenic Th1 cells. Moreover, oxLDL-C epitopes stimulate the production of oxLDL-C-specific autoantibodies, predominantly of the IgG2a isotype, which is Th1-associated. Elevated IgG titers correlate with an increased risk of ASCVD, underscoring their association with adverse prognoses [72].

Autoantibodies against MDA-modified lysine, a prominent oxLDL-C epitope, are found in various species, and immune complexes of these autoantibodies and oxLDL-C have been identified within atherosclerotic plaques [73]. Despite this, the exact role of these autoantibodies in atherogenesis remains unclear. Palinski et al. first explored their potential by immunizing LDL receptor-deficient (LDLr−/−) rabbits with homologous MDA-modified LDL-C particles. This approach elicited high titers of specific IgG antibodies and significantly reduced the extent of aortic atherosclerotic lesions [74]. Subsequent studies in rabbits and mice demonstrated similar outcomes, with LDL-C-based vaccines inducing significant IgG responses and corresponding reductions in lesion sizes, further supporting their atheroprotective potential [75,76]. Recent data also suggest that these humoral responses are accompanied by the expansion of memory and germinal center B cells, highlighting their adaptive immunological basis [77]. Interestingly, this evidence contrasts with findings linking elevated IgG titers to poor cardiovascular outcomes [13].

Ameli and colleagues were among the first to compare the effects of immunization with native LDL-C and oxLDL-C on atherosclerosis in hypercholesterolemic rabbits. Immunization with LDL-C and oxLDL-C reduced proximal aortic lesions by 74% and 48%, respectively, though reductions in other aortic regions were less pronounced. Both immunization protocols resulted in comparable increases in anti-oxLDL-C antibody titers. The authors hypothesized that native LDL-C might have undergone oxidation during preparation or immunization, and the lack of correlation between antibody levels and plaque areas suggested that cellular immune responses predominantly mediated the observed atheroprotection [78]. Similar results were observed in LDLr−/− mice, where native LDL-C and oxLDL-C immunization yielded comparable lesion size reductions, but only the oxLDL-C group exhibited elevated antibody levels, highlighting the critical role of cellular immunity [79]. Further evidence indicates that these responses are T cell-dependent, with antibody production to oxidized lipoproteins and plaque antigens requiring T cell involvement [80].

Beyond strategies to enhance IgG antibody titers, a significant body of research has focused on inducing mucosal tolerance to oxLDL-C. Tolerogenic vaccination approaches, initially explored in diseases such as multiple sclerosis, have also shown promise in atherosclerosis [81]. For instance, oral administration of oxLDL-C significantly reduced atherogenesis in LDLr−/− mice, accompanied by increased populations of CD4^+^CD25^+^FOXP3^+^ Tregs in the spleen and mesenteric lymph nodes. These Tregs responded to oxLDL-C with enhanced TGF-β production. In contrast, oral tolerance to MDA-modified LDL-C did not impact atherogenesis [82]. Similarly, nasal administration of oxLDL-C ameliorated atherosclerosis progression in ApoE−/− mice by increasing Tregs and upregulating TGF-β while suppressing pro-inflammatory Th1, Th2, and Th17 responses [83]. Another approach involving oxLDL-C-pulsed tolerogenic DCs in LDLr−/− mice also yielded atheroprotection. Habets et al. reported a groundbreaking approach to atherosclerosis immunomodulation through vaccination with bone marrow DCs pulsed with copper-oxLDL-C. In LDLr-deficient mice, the administration of these mature DCs (mDCs) three times prior to inducing atherosclerosis via a Western-type diet resulted in a remarkable 87% reduction in carotid artery lesion size and significant improvements in plaque stability. Notably, mDCs pulsed with an unrelated antigen, ovalbumin, showed no impact on lesion size or stability. The therapeutic effect was accompanied by the generation of oxLDL-C-specific T cells characterized by a reduced Th1 profile and increased levels of oxLDL-C-specific IgG, contributing to decreased foam cell formation [84].

Currently, two main mechanistic approaches have emerged for oxLDL-C-targeting vaccines, one aimed at increasing oxLDL-C-specific IgG antibody titers and another focused on promoting immune tolerance through Treg induction. Despite their seemingly divergent mechanisms, both approaches have demonstrated similar atheroprotective outcomes. These findings emphasize the need for further investigation in preclinical and clinical settings to elucidate the precise immunological roles of these vaccines and their potential implications for ASCVD prevention in humans.

#### 6.1.2. Apolipoprotein B100 (ApoB100)

Apolipoprotein B-containing lipoproteins, such as LDL-C and very low-density lipoprotein cholesterol (VLDL-C), play central roles in the initiation and progression of atherosclerosis. Among them, ApoB100 is integral to these processes, particularly due to its capacity to acquire immunogenic properties when modified by oxidative products like lysine and histidine residues on oxLDL-C particles [85]. Fredrikson et al. identified 102 immunogenic epitopes within ApoB100 through a peptide library screening, revealing their capacity to provoke humoral responses in humans. Modified forms, such as those altered by MDA, have been a focal point in developing ApoB100-targeting atherosclerosis vaccines [86].

Immunization studies using ApoB100-derived peptides have demonstrated substantial reductions in atherosclerosis in preclinical models. For instance, ApoE−/− mice vaccinated with MDA-modified peptides, including p143 and p210, showed an approximately 60% lesion reduction and increased lesion collagen content. These effects were mediated through specific IgG antibody responses [87]. Similarly, vaccination with native human ApoB peptides resulted in robust humoral responses and atheroprotection, though not all peptides (e.g., peptide-1) were effective [88]. Notably, MDA-modified peptides 45 and 74 promoted not only lesion reduction but also a shift in the immune response from a Th1-dominated profile (IgG2a isotype) to a Th2 phenotype (IgG1 isotype), indicating a role for T cell modulation [89]. This immunomodulation was further supported by studies showing reductions in plaque areas without concurrent increases in specific antibody levels, suggesting that cellular immunity, including Tregs, plays a vital role in the protective effects of ApoB100-targeting vaccines [90].

A growing body of evidence implicates Tregs in the anti-atherogenic effects of ApoB100 vaccines. Vaccination strategies using MHC-II-restricted ApoB epitopes have demonstrated lesion size reductions alongside Treg expansion, increased IL-10, and decreased IFN-γ and IL-17 levels [91,92,93,94]. Mailer et al. provided further insights by transferring naïve CD4^+^ T cells recognizing ApoB100 into transgenic mice. The transfer induced tolerance characterized by an increase in Treg type 1 cells that suppress ApoB100-specific responses via IL-10 [95]. However, Tregs themselves can acquire pro-inflammatory phenotypes (Th17/Th1) during disease progression, suggesting that therapeutic strategies targeting Treg stability must be tailored to disease stages [96].

In parallel to tolerogenic strategies, delivery methods have been refined to enhance the efficacy of ApoB100-based vaccines. For example, intranasal immunization with peptide p210 in ApoE−/− mice expanded antigen-specific splenic Tregs and suppressed effector T cell responses upon rechallenge [97]. Subcutaneous injection of the same peptide induced similar Treg expansion and atheroprotection, with anti-CD25 antibody treatment reversing these benefits and confirming Treg-mediated protection [98]. More recently, active immunization with the ApoB100 peptide p210 has shown promise in reducing experimental atherosclerosis, and a nanoparticle-based delivery system was explored to advance this strategy toward clinical application. Using peripheral blood mononuclear cells (PBMCs) from patients with ASCVD, P210 was found to provoke T cell activation and memory responses. P210 was then formulated into self-assembling peptide amphiphile micelles (p210-PAMs), which were effectively taken up by dendritic cells and co-localized with MHC-I molecules, supporting their potential as a vaccine. In ApoE−/− mice, p210-PAM immunization suppressed p210-specific CD4^+^ T cell proliferation and CD8^+^ T cell cytolytic responses, modulated macrophage polarization, and significantly reduced aortic atherosclerosis [99].

An alternative approach involves tolerogenic DCs pulsed with ApoB100 and IL-10. When administered to LDLr−/− mice expressing human ApoB100, this strategy reduced aortic atheroma size, decreased systemic inflammation, and induced Tregs while suppressing IFN-γ and other pro-inflammatory responses [100]. Emerging evidence also suggests a role for CD8^+^ T cells in ApoB100 vaccine-mediated atheroprotection. Immunization with p210 in ApoE−/− mice upregulated CD8^+^ T cells, while adoptive transfer of these cells into naïve mice provided protective effects. The suppression of DCs at plaques and vaccination sites suggests interplay between adaptive and innate immunity [101]. Additionally, CD8^+^CD25^+^ T cells, identified as having a suppressive phenotype, were shown to repress splenic CD4^+^ T cells and reduce atherosclerosis in recipient mice [102].

#### 6.1.3. Apolipoprotein CIII (ApoCIII)

Apolipoprotein CIII (ApoCIII), a key component of triglyceride-rich lipoproteins such as chylomicrons, VLDL-C, and cholesterol remnants, plays a crucial role in lipid metabolism and atherosclerosis development. It contributes to elevated plasma TG levels through multiple mechanisms, including the inhibition of lipoprotein lipase activity and decreased hepatic clearance of TG-rich lipoproteins. Furthermore, ApoCIII promotes the adhesion of LDL-C particles to intimal proteoglycans, highlighting its significant proatherogenic properties [103]. Genetic evidence strongly supports the role of ApoCIII in CV health. The overexpression of ApoCIII is associated with hypertriglyceridemia and atherogenesis, whereas loss-of-function mutations result in reduced TG levels and lower CVD risk [104]. These findings have made ApoCIII an attractive therapeutic target for managing hypertriglyceridemia and related CV conditions.

Therapeutic approaches targeting ApoCIII have shown significant potential, particularly the use of antisense oligonucleotides (ASOs) designed to reduce its expression. These interventions have demonstrated substantial reductions in plasma TG levels, providing a promising avenue for treating hypertriglyceridemia and mitigating its associated risks [105,106]. In addition to ASOs, the feasibility of an ApoCIII-targeting vaccine has also been explored. In 2017, Chackerian et al. evaluated a virus-like particle (VLP)-based vaccine in murine models. The study demonstrated a sustained reduction in ApoCIII levels, accompanied by a 30–40% decrease in plasma TG concentrations [107].

Despite these promising findings, significant gaps remain in understanding the broader implications of ApoCIII-targeting therapies. Notably, the direct effects of ApoCIII reduction on atherosclerosis progression and ASCVD risk have not been thoroughly investigated, even in preclinical settings. Furthermore, questions regarding the long-term safety, immune responses, and mechanistic impacts of such interventions need to be addressed. For instance, it is crucial to explore how the modulation of ApoCIII influences systemic inflammation, arterial wall biology, and plaque stability, as these factors are integral to atherosclerosis pathophysiology.

### 6.2. Vaccination Formulas Against Proteins Modulating Lipoprotein Metabolism

#### 6.2.1. Proprotein Convertase Subtilisin/Kexin Type 9 (PCSK9)

Proprotein convertase subtilisin/kexin type 9 (PCSK9) is a serine protease integral to cholesterol metabolism. It regulates the availability of LDLr by binding to their extracellular epidermal growth factor-like repeat A (EGFA) domain, leading to receptor internalization and lysosomal degradation. This inhibits LDLr recycling to the cell surface, resulting in elevated circulating LDL-C levels. Gain-of-function mutations in PCSK9 are linked to familial hypercholesterolemia (FH), while loss-of-function mutations correlate with lower LDL-C levels and reduced CV event rates [108]. Growing evidence suggests that PCSK9 plays a broader role beyond lipid metabolism, particularly in inflammation and immunity, by regulating both innate and adaptive immune cells and inflammatory responses [109]. It has also been studied in autoimmune conditions such as rheumatoid arthritis, systemic lupus erythematosus, and multiple sclerosis [110]. These insights indicate that targeting PCSK9 could provide therapeutic benefits beyond cholesterol management. However, further studies are needed to clarify its role in immune regulation and its potential as a therapeutic target in autoimmune diseases.

To mitigate the pro-atherogenic effects of PCSK9, therapeutic strategies have focused on reducing its activity or expression. Monoclonal antibodies, such as evolocumab and alirocumab, have been approved as adjunct treatments for hypercholesterolemia and ASCVD, demonstrating efficacy in lowering LDL-C and improving cardiovascular outcomes. However, challenges like cost and lifelong dosing requirements limit their broader use [111,112,113]. Inclisiran, an siRNA targeting PCSK9 mRNA, offers an alternative, achieving prolonged LDL-C reductions through a biannual dosing regimen. Other emerging strategies include antisense oligonucleotides and clustered regularly interspaced short palindromic repeats (CRISPR)-based gene-editing approaches, which have shown promise in preclinical studies [113,114,115].

Encouraging results from these therapies have spurred interest in PCSK9-targeting vaccines, which could provide long-lasting LDL-C reduction without frequent dosing. Several vaccine formulations have demonstrated success in preclinical models. VLP vaccines conjugate PCSK9 peptides to bacteriophage Qβ particles or genetically fuse PCSK9 sequences to bacteriophage MS2 coat proteins. Studies have shown high peptide-specific IgG titers, reduced serum PCSK9 levels, and upregulated LDLr activity, leading to decreased LDL-C and atherosclerotic lesion size [116,117,118,119]. Peptide-based vaccines involve PCSK9 epitope peptides combined with adjuvants like tetanus toxoid (TT) or encapsulated in nanoliposome (NP) carriers [120,121]. To address the high cost and frequent dosing required with passive immunization, a nanoliposomal PCSK9-specific active vaccine was developed and evaluated in BALB/c mice. The vaccine, termed L-IFPTA+, utilized a peptide construct (IFPT) conjugated to NPs and formulated with an alum adjuvant. Mice vaccinated with L-IFPTA+ demonstrated the highest IgG antibody titers against PCSK9, with sustained humoral responses observed over 48 weeks post-prime vaccination. The antibodies effectively inhibited the PCSK9-LDLr interaction and reduced plasma PCSK9 levels. Immunogenic safety analysis revealed increased anti-inflammatory CD4^+^ Th2 cells and IL-4 production without affecting inflammatory Th1 or cytotoxic CD8^+^ T cells [121]. A recent study using ferritin as an adjuvant reported similar immunogenicity and efficacy [122].

Preclinical studies in mice and non-human primates have consistently demonstrated favorable safety profiles and durable antibody responses, with significant reductions in LDL-C and atherosclerosis progression [123,124]. The first clinical trial of PCSK9 vaccines assessed safety and immunogenicity but has yet to publish results. A subsequent phase I trial evaluated two peptide vaccines, AT04A and AT06A. Both were safe and immunogenic, but only AT04A significantly lowered LDL-C, warranting further development ([125], NCT02508896). Despite promising results, limitations persist. A recent study highlighted the inability of a PCSK9-TT vaccine to protect against acute inflammation and oxidative stress in mice. Additionally, further exploration is needed to refine vaccine formulations, assess long-term effects, and optimize delivery methods [126,127].

#### 6.2.2. Cholesteryl Ester Transfer Protein (CETP)

Cholesteryl ester transfer protein (CETP) is a hepatic glycoprotein central to lipid metabolism and atherosclerosis development. It binds to high-density lipoprotein cholesterol (HDL-C) particles, mediating the transfer of cholesteryl esters, TGs, and phospholipids from HDL-C to proatherogenic ApoB-containing lipoproteins such as LDL-C, VLDL-C, and lipoprotein(a) [Lp(a)]. While CETP facilitates reverse cholesterol transport, an atheroprotective process, its predominant role is proatherogenic. CETP activity reduces plasma HDL-C levels, increases LDL-C and VLDL-C concentrations, and enhances atherogenesis, as demonstrated in transgenic mice expressing the human CETP gene [128]. Conversely, transient inhibition of CETP in animal models, using monoclonal antibodies, small molecules, or antisense oligonucleotides, results in elevated HDL-C levels and reduced atherosclerosis progression [129]. Beyond CETP’s well-known function in lipid metabolism and transport, recent studies highlight its involvement in the innate immune system, particularly in the efficient hepatic sequestration and elimination of lipopolysaccharides (LPSs), a key component of the outer membrane of Gram-negative bacteria [130]. A recent study also demonstrated that the pharmacological inhibition of CETP enhanced monocyte activation and bacterial clearance and reduced *Streptococcus pneumoniae*–associated mortality in mice [131]. The involvement of CETP in both lipid transport and immune defense illuminates its multifaceted nature, although it also complicates the therapeutic targeting of CETP, especially in cardiovascular diseases.

In clinical settings, CETP inhibitors like anacetrapib and obicetrapib have shown favorable effects on lipid profiles by raising HDL-C and lowering LDL-C levels. However, these agents have not been approved for therapeutic use, as their CV benefits remain unclear and safety concerns persist. This uncertainty has motivated the exploration of alternative strategies, including CETP-targeting vaccines, to modulate CETP activity and prevent atherosclerosis. The first CETP-targeting vaccine combined CETP with TT and was administered intramuscularly in New Zealand White rabbits. This vaccine increased HDL-C, decreased LDL-C levels, and significantly reduced aortic lesion areas [132]. Subsequent studies expanded on this concept, using various CETP-derived epitopes in combination with antigens such as TT, hepatitis B virus core (HBc) particles, and asparaginase. These vaccines have consistently demonstrated atheroprotective effects in rabbits, mice, and pigs. The observed benefits include elevated anti-CETP antibody titers, reduced CETP activity, increased HDL-C levels, lowered LDL-C concentrations, and diminished atherosclerotic lesion size. A particularly interesting study involved an oral TT/CETP vaccine in rabbits, which induced the upregulation of atheroprotective cytokines IL-10 and TGF-β while simultaneously reducing pro-atherogenic cytokines such as TNF-α and IFN-γ [133].

The only clinical trial to date testing a CETP-targeting vaccine was an open-label, non-randomized, phase I study, conducted between May 2011 and July 2012. This trial evaluated the safety, immunogenicity, and dose response of a vaccine (ATH03) in healthy male participants with HDL-C levels ≤80 mg/dL. Although the trial has concluded, its results have not been published, leaving a gap in the understanding of the clinical potential of CETP-targeting vaccines (NCT01284582).

#### 6.2.3. Angiopoietin-like Protein 3 (ANGPTL3)

**Figure 2:**
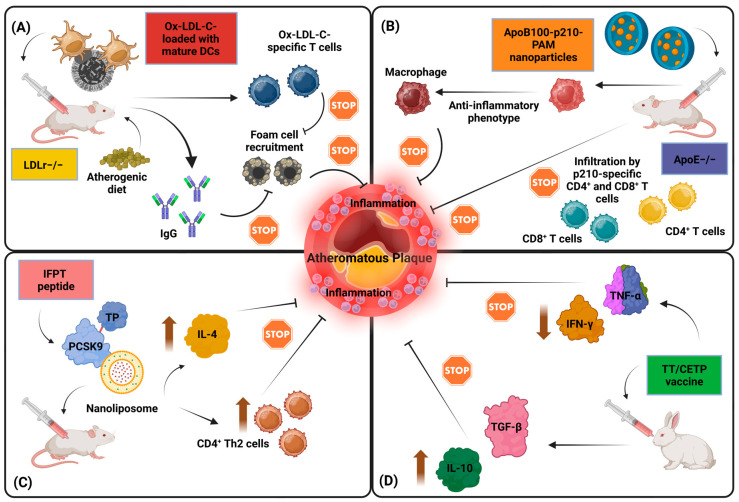
Experimental vaccine models targeting lipids to reduce inflammation in atherosclerosis. This figure depicts four distinct experimental vaccine strategies designed to mitigate the inflammation and progression of atherosclerosis by modulating immune responses. (**A**) OxLDL-C-pulsed dendritic cell transfer: LDLr−/− mice were treated with mature DCs pulsed with oxLDL-C before atherosclerosis induction via Western-type diet feeding. This approach promoted oxLDL-C-specific T cells and IgG production, diminishing foam cell recruitment and inflammation [84]. (**B**) PAM nanoparticle vaccine: PAM nanoparticles were used to deliver the p210 peptide in ApoE−/− mice models. This vaccine suppressed CD4^+^ and CD8^+^ effector T cells and shifted macrophage phenotypes, collectively reducing atherosclerotic burden and showing translational potential [99]. (**C**) Nanoliposome-based PCSK9-TP vaccine: A nanoliposome vaccine conjugated with PCSK9 and tetanus-derived peptides (IFPT peptide) was tested in atherosclerotic mice. The vaccine stimulated anti-inflammatory CD4^+^ Th2 cells and IL-4 secretion, promoting atheroprotective immune responses [121]. (**D**) Oral TT/CETP vaccine in rabbits: A combined oral vaccine targeting TT and CETP upregulated anti-inflammatory cytokines IL-10 and TGF-β while suppressing pro-inflammatory cytokines TNF-α and IFN-γ [133]. Abbreviations: ApoE: apolipoprotein E; CETP: cholesteryl ester transfer protein; DC: dendritic cell; IFN-γ: interferon-gamma; IL: interleukin; LDLr: low-density lipoprotein receptor; oxLDL-C: oxidized low-density lipoprotein cholesterol; PAM: poly(amino acid)-based; PCSK9: proprotein convertase subtilisin/kexin type 9; TGF-β: transforming growth factor-beta; Th2: T-helper type 2; TNF-α: tumor necrosis factor-alpha; TP: tetanus peptide; TT: tetanus toxoid. Created in BioRender. Kounatidis, D. (2024) https://BioRender.com/f60u021, assessed on 24 November 2024.

Angiopoietin-like protein 3 (ANGPTL3) is a soluble hepatic protein that plays a key role in lipid metabolism by inhibiting lipoprotein lipase (LPL), leading to increased plasma TG levels. Loss-of-function mutations in ANGPTL3 are associated with significant reductions in LDL-C, TGs, and CV risk, making it a compelling target for therapeutic and preventive interventions, including atherosclerosis vaccines. Preclinical studies have demonstrated the potential of ANGPTL3-targeting vaccines. In mouse models, two vaccine formulations effectively upregulated LPL activity, reduced serum TGs and LDL-C levels, and notably decreased atherosclerotic lesion sizes [134,135]. A follow-up study further emphasized the long-term efficacy of ANGPTL3 vaccination. Booster doses administered over the lifespan of mice maintained suppressed circulating TG levels without significant adverse effects, highlighting the durability and safety of this approach [136]. Figure 2 illustrates four experimental vaccine models designed to target lipids and reduce inflammation and progression of atherosclerosis.

## 7. Non-Lipid-Related Vaccines

### 7.1. Heat Shock Proteins

Heat shock proteins (HSPs) are a family of proteins present in both prokaryotic and eukaryotic organisms, whose primary role is to protect cells from stress by maintaining cellular proteostasis [137]. Under various stress conditions, HSPs perform chaperone functions by stabilizing the folding of newly synthesized proteins, facilitating the refolding of damaged proteins and inducing the degradation of misfolded ones. Furthermore, they play critical roles in cell signaling, cell cycle regulation, and apoptosis [138].

It is well established that humans have evolved both humoral and cellular immunity against microbial HSPs, particularly HSP65, which shares homology with human HSP60 [139]. However, in response to cellular stress induced by traditional CV risk factors such as hypertension, hypercholesterolemia, and smoking, mitochondrial expression of HSP60 in endothelial cells is significantly upregulated. This upregulation renders HSP60 a target of pre-existing humoral and cellular immunity [140]. During the progression of atherosclerosis, anti-HSP60 antibodies mediate complement activation and antibody-dependent cellular cytotoxicity (ADCC), leading to endothelial cell lysis. Additionally, extracellular HSP60 (exHSP60) triggers the production of pro-inflammatory cytokines such as TNF-α and IL-6, thereby perpetuating the inflammatory cascade [139].

The well-documented role of HSP65/60 cross-reactivity in the induction of autoimmune responses that drive atherosclerosis has spurred efforts to develop vaccines aimed at restoring immune tolerance to HSP65/60. Research teams have investigated both oral and nasal vaccine formulations, primarily utilizing mycobacterial HSP60 and HSP65 as immunogenic antigens [141,142,143,144]. Remarkably, these studies consistently demonstrated the induction of immune tolerance, marked by significant reductions in atherosclerotic plaque size. These reductions were accompanied by the expansion of CD4^+^CD25^+^FOXP3 Treg cells, the upregulation of atheroprotective cytokines such as TGF-β and IL-10, and a decrease in pro-atherogenic IFN-γ levels. Some studies also reported improvements in lipid profiles [145,146,147].

Similar results were observed with nasal immunization using recombinant *Helicobacter pylori* HSP60. This approach not only demonstrated atheroprotection but also correlated with the expansion of CD4^+^CD25^+^GARP^+^ Treg cells, which suppress Th1 and Th17 responses, as well as CD4^+^CD25- LAP^+^ Treg cells [148,149]. Notably, the subcutaneous administration of HSP60 in ApoE(−/−) mice failed to elicit atheroprotection, whereas oral administration of the same vaccine led to a marked reduction in plaque size at the aortic root. This effect was accompanied by the upregulation of myeloid-derived suppressor cells (MDSCs) [150].

### 7.2. Interleukins

The pivotal role of cytokines, particularly interleukins, in atherogenesis has identified them as promising targets for atherosclerosis vaccines. Research has primarily focused on IL-12, IL-15, and IL-1α. Given IL-12′s critical role as a key driver of the Th1-cell cytokine response, Hauer et al. investigated the effects of an IL-12-targeted vaccine in LDLr−/− mice. Their study demonstrated that vaccination induced anti–IL-12 antibodies, leading to a significant reduction in atheroma lesion areas and enhanced plaque stability. This was corroborated by an increased proportion of smooth muscle cells and collagen content in the neointima [151].

Similarly, DNA vaccination against IL-15 in LDLr−/− mice markedly reduced atherosclerotic lesion size. However, in contrast to IL-12 vaccination, it did not enhance lesion stability [152]. In another study, Tissot and colleagues showed that immunizing hypercholesterolemic mice with an anti–IL-1α Qβ-based vaccine significantly attenuated plaque progression in the descending aorta and aortic root. This effect was associated with reduced macrophage infiltration and lower expression levels of VCAM-1 and ICAM-1 [153].

More recently, two Qβ-based vaccines targeting interleukin-1 receptor type I (IL-1R1) demonstrated robust efficacy in reducing atherosclerotic plaque areas. These vaccines also promoted plaque stabilization and reduced macrophage infiltration in ApoE−/− mice. Furthermore, they significantly improved cardiac function and mitigated macrophage infiltration in C57BL/6J mice following MI, all while avoiding notable immune-mediated side effects [154].

### 7.3. Angiotensin II

In recent decades, the traditional understanding of the renin–angiotensin–aldosterone system (RAAS) as primarily responsible for extracellular volume and blood pressure regulation has been expanded to include its role in promoting inflammation, with angiotensin II identified as a key pro-inflammatory mediator. Angiotensin II elevates blood pressure through vasoconstriction and aldosterone secretion, which collectively stimulate arterial VSMC growth, induce oxidative stress within the intima, and recruit pro-inflammatory cytokines. These mechanisms position angiotensin II as a compelling target for the development of vaccines against atherosclerosis [155,156].

Since the early 2000s, various vaccine formulations targeting peptides of angiotensin II or the angiotensin II type 1 receptor (AT1R) have been tested in hypertensive rat models, consistently demonstrating substantial blood pressure-lowering effects across studies. Building on these preclinical successes, one phase I and one phase II clinical trial evaluated the VLP-based vaccine CYT006-AngQb. These trials reported encouraging reductions in blood pressure among healthy and hypertensive human participants, critically without significant safety concerns [157,158].

In 2016, further progress was achieved with a VLP-conjugated vaccine targeting AT1R. This study was the first to demonstrate atherosclerosis mitigation in hypercholesterolemic mice. Vaccination resulted in significant reductions in aortic plaque size, accompanied by marked decreases in adhesion molecule expression and suppressed macrophage accumulation, highlighting its potential as a dual-action therapeutic targeting both hypertension and atherosclerosis [159].

### 7.4. ADAMTS-7

A disintegrin and metalloproteinase with thrombospondin type 1 motif-7 (ADAMTS-7) is an extracellular matrix protease whose genetic locus has been identified through genome-wide association studies to be significantly correlated with CAD [160]. Although its precise physiological role in vascular biology remains incompletely elucidated, experimental evidence suggests that ADAMTS-7 promotes VSMC migration and subsequent neointima formation by degrading its primary substrate, cartilage oligomeric matrix protein (COMP) [161]. Additionally, ADAMTS-7 inhibits endothelial cell proliferation and migration through thrombospondin-1 degradation, thereby impeding the re-endothelialization of injured arteries and further contributing to neointima formation [162]. Recent findings have highlighted the role for ADAMTS-7 in destabilizing atherosclerotic plaques by reducing the inhibitory capacity of the tissue inhibitor of metalloproteinase-1 (TIMP-1) on matrix metalloprotease-9 (MMP-9), a protease linked to collagen degradation and CAD progression [163,164]. Conversely, studies in ADAMTS-7-deficient mice have demonstrated the mitigation of neointima formation and further evidence of atheroprotection [162,165,166].

Ma et al. designed three potential vaccines targeting ADAMTS-7, based on distinct B cell epitopes located within its catalytic domain and C-terminal thrombospondin repeats. Among these candidates, the vaccine targeting the catalytic domain, named ATS7vac, emerged as the most promising. ATS7vac significantly reduced atherosclerotic lesions in hyperlipidemic mice, decreased neointima formation in murine wire injury models, and alleviated all in-stent restenosis without notable safety concerns [167]. This outcome aligns with recent findings that the catalytic domain is critical in the pro-atherogenic effects of ADAMTS-7 [168].

Mechanistically, ATS7vac stimulated the production of specific antibodies against ADAMTS-7, effectively inhibiting its degradation of COMP and thrombospondin-1. This, in turn, suppressed VSMC migration and promoted endothelial repair through enhanced re-endothelialization. Unlike previous anti-atherosclerotic vaccines, which often target immune system components, ADAMTS-7 vaccines act on non-immune molecular pathways [167]. Despite promising preclinical results, further research is necessary to elucidate ADAMTS-7′s downstream targets and confirm the safety and efficacy of these vaccines in human populations at risk for atherosclerosis and its complications.

**Figure 3:**
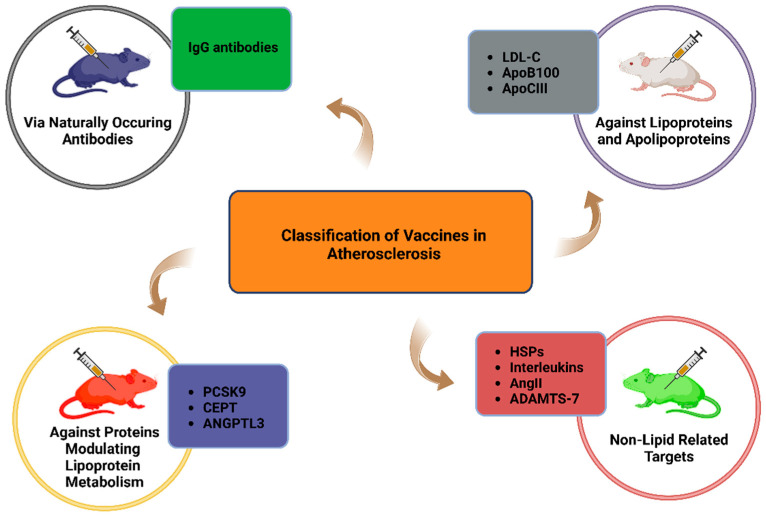
Primary vaccination targets in atherosclerosis. Abbreviations: ADAMTS-7: A disintegrin and metalloproteinase with thrombospondin motifs 7; ANGII: angiotensin II; ANGPTL3: angiopoietin-like protein 3; ApoB100: apolipoprotein B100; ApoCIII: apolipoprotein CIII; CETP: cholesteryl ester transfer protein; HSP: heat shock protein; IgG: immunoglobulin G; LDL-C: low-density lipoprotein cholesterol; PCSK9: proprotein convertase subtilisin/kexin type 9. Created in BioRender. Kounatidis, D. (2024) https://BioRender.com/f98n410, assessed on 24 November 2024.

Table 3 presents a summary of vaccine-based approaches and monoclonal antibody interventions aimed at preventing atherosclerosis and CVD. Additionally, Figure 3 illustrates the primary vaccination targets in atherosclerosis.

## 8. Multi-Target Vaccines

Beyond individual vaccines, a variety of multi-target vaccine formulations have been developed, offering a more comprehensive approach to atherosclerosis prevention. Most multitarget designs combine peptides from ApoB and HSP60, which have shown substantial atheroprotective effects. These effects are attributed to both humoral responses and immune tolerance induction, primarily mediated by Treg expansion. Notably, ApoB/HSP60 combination vaccines have demonstrated superior efficacy in reducing atherosclerotic lesion size compared to vaccines targeting ApoB or HSP60 individually [169,170].

Further enhancements have been achieved by incorporating *Chlamydia pneumoniae* antigens into ApoB100 and HSP60 vaccines. These antigens are recognized for their CTL-activating potential, primarily through interaction with MHC II molecules [171]. Studies utilizing these formulations have shown robust atheroprotection linked to immune tolerance via increased Treg populations [172,173]. Another innovative approach involved DCs pulsed with ApoB100 and IL-10. This combination achieved a remarkable 70% reduction in aortic lesion area, underscoring a strong protective effect against plaque formation [100].

In a more recent strategy, a trivalent VLP vaccine incorporating peptides from ApoB, CETP, and PCSK9 conjugated to bacteriophage Qβ-VLPs elicited high titers of antigen-specific IgG antibodies. This formulation inhibited all three targets simultaneously and significantly reduced total cholesterol levels [174]. Additionally, a fusion protein vaccine combining HSP65, CETP, PADRE (a universal T cell epitope), and tetanus toxoid (TT) was administered intranasally to male New Zealand white rabbits. This vaccine provided notable atheroprotection, accompanied by reductions in LDL-C and IFN-γ, alongside an upregulation of the anti-inflammatory cytokine IL-10 [175].

These multi-target strategies underscore a multifaceted approach to atherosclerosis management, leveraging immune modulation, lipid metabolism regulation, and inflammation control to achieve synergistic therapeutic outcomes.

## 9. Overcoming Translational Challenges in Developing Atherosclerosis Vaccines for Clinical Application

Vaccination stands as one of the most transformative achievements in medical history, successfully preventing bacterial and viral infections [176]. Recent advancements reveal that vaccines could also modulate immune responses in autoimmune conditions like diabetes mellitus and multiple sclerosis, primarily in preclinical models [177]. Likewise, progress in atherosclerosis vaccines has been promising at the preclinical level; however, studies on ApoB-specific T cells in humans remain scarce [94]. For the successful development of human atherosclerosis vaccines, foundational questions about immunity to ApoB/LDL-C and advancements in vaccine technology must be addressed.

### 9.1. Epitope Selection and Challenges in Immune Presentation

The effective presentation of peptide epitopes to CD4^+^ T cells depends on their binding to MHC-II molecules, known as the HLA complex in humans. For instance, HLA-DRB1*07:01 is a relatively common allele capable of binding and presenting the ApoB peptide p18 [94]. However, the high heterogeneity of HLA alleles poses a significant challenge to developing a universal atherosclerosis vaccine, as not all peptides bind all HLA types with high affinity [178]. Additionally, it remains unclear whether low- versus high-affinity peptides promote divergent T cell phenotypes, simultaneously influencing pro- and anti-inflammatory T cell clones [179]. The ideal scenario of a single epitope binding universally across all HLA types is rarely feasible.

A potential solution involves identifying a pool of immunogenic ApoB peptides through computational predictions and experimental validation. For instance, Wolf et al. identified 30 peptides capable of eliciting CD4^+^ T cell responses in vitro, both in healthy individuals and in patients with coronary heart disease [96]. However, translating these findings into mouse models has proven challenging due to limited sequence homology between humans and mice, with p18 being the only peptide identified to share such homology [96]. Other peptides may induce “foreign” immune responses in mice, skewing experimental outcomes. This underscores the need for the development of human HLA-transgenic mouse models with atherosclerosis-prone genetic backgrounds, a task that presents logistical complexities.

An innovative approach utilizing Ii-Key hybrid technology has shown promise. This technology, successfully tested in cancer vaccine trials, involves a fragment of the Ii protein (or MHC class II-associated invariant chain) that binds to all HLA types with high affinity, preventing endogenous peptide binding. Ii-Key also facilitates the presentation of linked antigenic peptides by displacing the invariant chain peptide (CLIP) from MHC class II molecules [180]. By linking MHC-II-restricted epitopes to Ii-Key, this method promotes CD4^+^ T cell activation without requiring HLA matching. Although promising, the application of Ii-Key technology in the context of atherosclerosis remains unexplored.

### 9.2. Determination of Appropriate Adjuvants, Carriers, and Routes of Administration

Adjuvants enhance immune responses to antigens by activating the innate immune system [181]. Their selection is influenced by the type of antigen, the desired immune response, and the route of administration. Several adjuvants have been used in preclinical studies of atherosclerosis vaccines, including Complete Freund’s Adjuvant (CFA), which, while broadly used in experimental settings and proven to be atheroprotective, is unsuitable for clinical use due to its toxicity [182]. Similarly, Incomplete Freund’s Adjuvant (IFA) has largely been discontinued due to safety concerns, although it continues to be studied. Aluminum-based adjuvants have been safely used in clinical settings for decades, while Cholera Toxin B Subunit (CTB) has demonstrated success in clinical trials [183]. The CFA-IFA prime-boost sequence has shown atheroprotective effects, whereas single vaccinations with CFA and the same antigen appear to exacerbate atherosclerosis [183,184]. Additionally, adjuvants such as IFA and aluminum have shown the ability to modulate immune responses independently of specific antigens, potentially by shifting immune responses toward regulatory or anti-inflammatory profiles [185,186].

Addavax, a novel squalene-based adjuvant resembling MF59 (used in influenza vaccines), has demonstrated strong atheroprotective effects in ApoE−/− mice [187]. Notably, an Addavax-ApoB peptide-6 vaccine reduced atherosclerotic lesion area similarly to the CFA/IFA vaccine but without inducing an antigen-specific antibody response, highlighting its potential for clinical translation despite the unclear protective mechanisms [93]. Additionally, adjuvant-free approaches are emerging, as demonstrated by Herbin et al., who showed that continuous low-dose delivery of ApoB peptide mixtures via subcutaneous minipumps effectively reduced atherosclerosis by inducing Treg responses [188].

Regarding carriers, liposomes have been well-established in clinical settings as reliable drug delivery systems, making them a promising option for atherosclerosis vaccine development [189]. Research has shown that ApoB peptides delivered via liposomes induce atheroprotection and antigen-specific Treg responses [190]. Similarly, VLPs have been tested as carriers for atherosclerosis-related peptides, particularly in PCSK9-targeting vaccines, with promising preclinical results [191].

Vaccines for atherosclerosis have been administered via nasal, oral, subcutaneous, intraperitoneal, and intramuscular routes. Subcutaneous and intraperitoneal methods are the most frequently used, eliciting robust humoral and cellular responses [192]. Alternatively, oral and nasal administration of specific antigens, such as HSPs or LDL-C-related antigens, have provided atheroprotection by expanding tolerogenic Tregs [193]. However, studies directly comparing mucosal and parenteral administration for preventing or treating atherosclerosis are lacking. While subcutaneous, intramuscular, and mucosal routes appear feasible for human application, the optimal route for vaccine delivery remains undetermined, necessitating further research.

### 9.3. Determination of the Optimal Vaccination Timing

Preclinical studies often administer vaccines to young, disease-free mice before introducing a Western-type diet, simulating an early prevention strategy. Translating this to humans would involve vaccinating children or adolescents before the onset of lifestyle-related risk factors, akin to infectious disease vaccination strategies. However, vaccinating individuals with established atherosclerosis presents unique challenges, particularly due to phenotypic shifts in Tregs during disease progression [194]. Under hypercholesterolemic conditions, ApoB^+^ Tregs lose FOXP3 expression and acquire proatherogenic characteristics by converting into Tfh, Th1, or Th17 cells [194,195].

The timing of immunization critically affects outcomes. For instance, an ApoB p6 vaccine was protective in younger mice but worsened atherosclerosis in older, pre-fed mice [184]. Conversely, vaccines targeting the p3 epitope did not accelerate disease under similar conditions [182]. Although delayed immunization has shown benefits in some cases [188], comparative studies evaluating various vaccination protocols (e.g., dosage, frequency) are limited. Furthermore, the hypothesis that periodic vaccinations could improve the long-term stability of atheroprotective immunity warrants further exploration.

### 9.4. Identification of Eligible Patients for Atherosclerosis Vaccination

Despite advances in lipid-lowering therapies, inflammation remains a significant residual risk factor for cardiovascular events, indicating the need for additional therapeutic strategies [196]. Immune activation, which is inadequately addressed by current therapies, may be mitigated by vaccines targeting immune pathways in atherosclerosis. Emerging techniques, such as restimulation assays to detect ApoB-specific T cells [197] and tetramer-based methods for their quantification [85], could aid in identifying individuals most likely to benefit from these strategies. Combining vaccination with lipid-lowering and anti-inflammatory treatments could target both inflammatory and immune-driven risks, paving the way for personalized cardiovascular care.

## 10. Future Perspectives in Atherosclerosis Vaccines: The Transformative Potential of Organ-on-a-Chip and Microphysiological System Technologies

Organ-on-a-chip (OoC) and microphysiological system (MPS) platforms represent cutting-edge advancements in biomedical research, functioning as microfluidic devices that replicate the structure and function of human tissues and organ systems. These technologies offer distinct advantages over traditional in vitro and animal testing methods by simulating complex biological processes in a controlled environment [198]. Concerning atherosclerosis, OoCs can recreate key aspects of the arterial structure, including endothelial cells, VSMCs, and the extracellular matrix. Moreover, these platforms can replicate fluid dynamics, such as shear stress, which is a critical factor in the development of atherosclerotic plaques [199]. By integrating immune cells (e.g., macrophages) and lipids into these systems, researchers can observe the real-time development of plaques, thereby providing a promising platform for evaluating the efficacy and toxicity of novel therapeutic interventions. Personalized OoCs, derived from patient-specific cells, further enhance the ability to study individual responses to treatment [199].

Beyond drug development, OoC platforms hold immense promise for advancing vaccine research against atherosclerosis. By replicating the intricate interactions of the cardiovascular and immune systems, these models may offer a human-relevant alternative for testing vaccine efficacy. Specifically, OoCs can mimic the formation and progression of atherosclerotic plaques, enabling the assessment of vaccine-induced changes in lipid deposition, foam cell formation, and smooth muscle cell behavior. The integration of immune cells and vascular endothelial cells allows researchers to evaluate how vaccines modulate immune responses within the vascular microenvironment, including their ability to promote anti-inflammatory or protective effects [200].

Furthermore, OoCs can facilitate mechanistic studies that refine vaccine design by targeting key processes such as LDL-C oxidation, immune cell recruitment, and pro-inflammatory cytokine production. By using patient-derived cells, these platforms also enable personalized assessments of vaccine efficacy, ensuring that treatments can be tailored to individual patient profiles. The potential of OoCs extends even further with the development of multi-organ-on-a-chip systems [201]. These integrated platforms may provide a holistic view of how vaccines targeting atherosclerosis interact with the entire body, revealing potential off-target effects or synergistic mechanisms. This comprehensive approach will be critical in addressing the complexities of vaccine development for a multifactorial disease like atherosclerosis.

A notable advancement in this field is the recent work by Jeger-Madiot et al., who developed a novel lymphoid organ-on-a-chip (LO-chip) model [202,203]. This system used a microfluidic chip seeded with human PBMCs embedded in a 3D collagen matrix to address challenges in predicting vaccine immunogenicity. The model successfully mimicked vaccine boosting by perfusing severe acute respiratory syndrome-coronavirus-2 (SARS-CoV-2) spike protein, which elicited amplified memory B cell responses, plasmablast differentiation, and spike-specific antibody production. Importantly, the platform demonstrated compatibility with mRNA vaccines delivered via lipid nanoparticles and enabled the comparison of monovalent versus bivalent vaccine responses. Such innovations highlight the potential of OoCs to transform vaccine development across a broad range of applications, including atherosclerosis [202].

## 11. Conclusions

Atherosclerosis is increasingly recognized as an immune-mediated condition in which pro-inflammatory responses exacerbate disease progression, while regulatory cells offer protective effects, underscoring their potential as therapeutic targets. However, the microenvironment significantly influences immune responses, occasionally altering protective mechanisms into pathogenic roles. Certain immune cells, such as CD8^+^ T cells, B1 cells, Th2 cells, and Th17 cells, exhibit context-dependent duality, necessitating further exploration of their specific functions. Residual CVD risk, despite optimal lipid control, has shifted focus toward anti-inflammatory and immune-modulating therapies. Among these, vaccination strategies against atherosclerosis hold particular promise. Passive immunization with naturally occurring IgG antibodies targeting oxLDL-C epitopes and active immunization to stimulate immune responses against antigens such as oxLDL-C, ApoB100, and PCSK9 have shown encouraging preclinical results. Notably, vaccines targeting oxLDL-C or ApoB100 demonstrate dual benefits of promoting atheroprotection and inducing immune tolerance to self-antigens. Despite these advancements, the translation of atheroprotective vaccines into clinical practice remains challenging. Key hurdles include optimizing adjuvants, epitopes, and delivery modalities to enhance efficacy, elucidating mechanisms underlying T cell phenotype switching to prevent adverse immune reactions and identifying optimal vaccination schedules. While these challenges are substantial, continued research and innovation hold the potential to revolutionize the management of ASCVD through the successful development of effective atheroprotective vaccines.

## Figures and Tables

**Table 1:** Key areas in the pathophysiology of atherosclerosis and immune response.

Key Areas	Main Points
The role of inflammation and immune activation [14,15,16,17]	- Inflammation is a core driver of atherosclerosis, triggering both innate and adaptive immune responses - Key innate immune cells like monocytes, macrophages, neutrophils, mast cells, and DCs produce cytokines and chemokines, activate phagocytosis, and initiate adaptive immunity - Adaptive immunity involves T cells, B cells, and ILCs, with a unique subset bridging the innate and adaptive immune systems - OxLDL-C serves as a pro-inflammatory agent in atherosclerosis
Atherosclerotic plaque development [18,19,20,21,22,23,24,25,26,27]	- Foam cell formation and atherosclerotic plaque development are key stages in atherogenesis - Resident and monocyte-derived macrophages clear aggregated OxLDL-Cs, transforming into foam cells - VSMCs also contribute to foam cell formation - Subendothelial cholesterol aggregation leads to cholesterol crystal formation, promoting inflammation - Early plaque stages include fatty streaks, while advanced plaques consist of a necrotic core and fibrous cap - Atheroma’s oxidative environment promotes pro-atherogenic immune responses
T cells and antigen presentation [28,29,30,31,32,33,34,35]	- T cells play a central role in atherogenesis through antigen presentation by APCs - Early in atherosclerosis, Tregs are prevalent, but they shift to CD4^+^ effector Th cells as the disease progresses - Tregs maintain immune tolerance, whereas Th cells drive inflammation and plaque formation - Regulatory and effector T cells influence immune modulation through cytokines and co-stimulatory factors - APCs present antigens to T cells, driving polarization and promoting the development of atherosclerotic lesions
Th cell polarization, cytotoxic T cells, and B cells [36,37,38,39,40,41]	- Th1 cells in plaques promote inflammation by producing cytokines like IFN-γ and TNF-α, driving foam cell formation - Th2 cells, which produce IL-4, IL-5, and IL-13, may play an atheroprotective role by fostering collagen deposition and reducing inflammation - Th17 cells show context-dependent effects, with both pro-atherogenic and anti-atherogenic properties - Cytotoxic CD8^+^ T cells promote apoptosis and inflammation, whereas B cells in plaques contribute to both immune responses, with B1 cells providing an atheroprotective role and B2 cells potentially exacerbating atherosclerosis

Abbreviations: APC: antigen-presenting cell; CD8^+^: cytotoxic T cells; DC: dendritic cell; IFN-γ: interferon gamma; IL: interleukin; ILC: innate lymphoid cell; OxLDL-C: oxidized low-density lipoprotein cholesterol; Th: T helper; TNF-α: tumor necrosis factor-alpha; Tregs: regulatory T cells; VSMC: vascular smooth muscle cell.

**Table 2:** Key preclinical and clinical studies of immunomodulating agents against atherosclerosis.

Author, Year	Agent	Purpose of the Study	Results of the Study	Conclusions
Santos Zas, 2021 [54]	CD8^+^-specific monoclonal antibody (CD8 mAb)	- To understand the contribution of CD8^+^ T cells and their cytotoxic effector molecule, granzyme B, on adverse cardiac remodeling after MI in mice - To investigate the effect of CD8^+^ T cell depletion by CD8 mAb on myocardial injury	- CD8^+^ T cells infiltrate the infarcted myocardium early after MI, with granzyme B mediating tissue damage - Mice receiving CD8 mAb, exhibited reduced cardiac dysfunction, smaller infarct sizes, and improved post-MI survival compared to controls	Targeting CD8^+^ T cells may offer novel therapeutic strategies to improve outcomes in patients with ischemic heart disease
Rurik, 2022 [55]	In vivo generation of antifibrotic CAR T cells by delivering modified mRNA in T cell–targeted LNPs	To test the hypothesis that in vivo generated CAR T cells targeting specific cardiac fibroblasts could mitigate pathological remodeling and improve cardiac function following injury in mice	- CAR T cells significantly decreased fibrosis in the heart tissue and enhanced cardiac function - There were minimal off-target effects, highlighting the specificity of the therapy for cardiac fibroblasts	In vivo-generated antifibrotic CAR T cells offer a potential new treatment strategy against ischemic heart disease and atherosclerosis
Zhao, 2022 [57]	Rituximab	To evaluate the safety of rituximab administration in patients with acute STEMI	- Rituximab was generally well-tolerated, with a manageable safety profile - A marked reduction in inflammatory markers was observed in the rituximab group - Imaging showed a trend toward reduced infarct size and improved left ventricular function, although statistical significance was not consistently achieved in the small sample size	Rituximab appears to be safe for use in patients with acute STEMI, providing a potential avenue for inflammation-targeted therapy
Ridker, 2017 [10]	Canakinumab	To explore whether three different doses of canakinumab (50, 100 and 300 mg) versus a placebo could improve CV outcomes in MI survivors with a hsCRP level of at least 2 mg/L	- Canakinumab reduced MACEs by 15% in patients treated with the 150 mg dose compared to the placebo (hazard ratio [HR]: 0.85, *p* = 0.007), with this dose showing the most significant benefits after statistical adjustments for multiplicity - Canakinumab had no effect on lipid parameters from baseline - Canakinumab-treated groups developed fewer fatal malignancies, particularly lung cancer - Canakinumab increased the risk of fatal infections or sepsis, particularly at higher doses - Leukopenia and mild infections were also more common in the treatment groups	- Canakinumab effectively reduced CV events and potentially incident lung cancer - These benefits must be weighed against the increased risk of infections
Tardif, 2019 [11]	Colchicine	To assess the safety and efficacy of low-dose colchicine (0.5 mg daily) versus a placebo on MACE reduction among patients with a recent MI	- Colchicine significantly reduced the primary composite endpoint of death from CV causes, resuscitated cardiac arrest, MI, stroke, or urgent hospitalization for angina leading to coronary revascularization (HR: 0.77; 95% confidence interval [CI] 0.61 to 0.96; *p* = 0.02) - Patients in the colchicine arm experienced more frequent episodes of diarrhea and pneumonia (not statistically significant)	In patients with a recent MI, low-dose colchicine was effective in reducing the risk of ischemic CV events compared to the placebo
Nidorf, 2020 [44]	Colchicine	To evaluate the efficacy and safety of low-dose colchicine (0.5 mg daily) versus a placebo in reducing CV events in patients with CAD	- The composite of CV death, MI, ischemic stroke, or ischemia-driven coronary revascularization occurred in 6.8% of patients in the colchicine group vs. 9.6% in the placebo group. Relative risk reduction (RRR): 31% (HR: 0.69; 95% CI: 0.57–0.83; *p* < 0.001) - Colchicine was generally well-tolerated, but mild gastrointestinal symptoms (e.g., diarrhea) were more frequent in the colchicine group - There was a slightly higher rate of non-CV death in the colchicine group (HR 1.51), although the causes were varied and not clearly attributable to colchicine	- Colchicine provides a clinically meaningful reduction in CV events in patients with stable CAD when added to standard therapies, such as statins and antiplatelets - The safety profile requires careful consideration, particularly in terms of non-CV mortality
Ridker, 2019 [50]	Methotrexate	To evaluate whether low-dose methotrexate (15 to 20 mg weekly) could reduce the risk of CVD events in patients with previous MI or multivessel CAD who additionally had either type 2 diabetes or metabolic syndrome	- Low-dose methotrexate did not significantly reduce the incidence of major cardiovascular events compared to placebo - Methotrexate did not also lower levels of key inflammatory markers, including CRP, IL-1β, or IL-6 - Methotrexate was connected to elevations in liver-enzyme levels, reductions in leukocyte counts and hematocrit levels, and an increased incidence of non-basal-cell skin cancers than placebo	Low-dose methotrexate, despite its well-established anti-inflammatory properties, is not effective for CV risk reduction
Ridker, 2021 [51]	Ziltivekimab	To assess the safety and efficacy of Ziltivekimab in reducing levels of inflammatory biomarkers like hsCRP, fibrinogen, and serum amyloid A, in patients with moderate to severe CKD, and hsCRP of at least 2 mg/L	- Ziltivekimab demonstrated a dose-dependent reduction in hsCRP - Dose-dependent reductions were also observed for fibrinogen, serum amyloid A, haptoglobin, secretory phospholipase A2, and lipoprotein(a) - Ziltivekimab reduced systemic inflammation without notable variability between patients - It did not affect the total cholesterol-to-HDL-C ratio - The treatment was generally well-tolerated with a manageable safety profile	Ziltivekimab showed promise in reducing chronic inflammation in CKD patients at high risk for CV events
Zhao, 2022 [52]	IL-2	To evaluate the impact of low-dose IL-2 therapy on Treg activation, expansion, and function in individuals with ischemic heart disease	- Low-dose IL-2 significantly increased the number and activity of Tregs in treated patients compared to baseline and placebo controls - The effect was sustained over the treatment period, with no significant activation of effector T cells	Low-dose IL-2 expanded Tregs and reduced systemic inflammation without adverse events of major concern

Abbreviations: CAD: coronary artery disease; CAR: chimeric antigen receptor; CD8^+^: cytotoxic T cells; CD8 mAb: CD8-specific monoclonal antibody; CI: confidence interval, CKD; chronic kidney disease; CRP: C-reactive protein; CV: cardiovascular; CVD: cardiovascular disease; HDL-C: high-density lipoprotein cholesterol; HR: hazard ratio; hsCRP: high-sensitivity CRP; IL-1β: interleukin-1β; IL-2: interleukin-2; IL-6: interleukin-6; LNPs: lipid nanoparticles; mAb: monoclonal antibody; MACEs: major adverse cardiovascular events; MI: myocardial infraction; mRNA: messenger ribonucleic acid; RRR: relative risk reduction; STEMI: ST-segment elevation myocardial infraction; Treg: T regulatory cells.

**Table 3:** Summary of vaccine-based approaches for atherosclerosis and cardiovascular disease prevention.

Author, Year	Vaccine Type	Purpose of the Study	Results of the Study	Conclusions
de Vries, 2021, [68]	Chimeric anti-PC (PC-mAb(T15)) and human monoclonal anti-PC (PC-mAbs)	To develop and evaluate the anti-inflammatory and anti-atherosclerotic properties of monoclonal antibodies targeting PC in CVD	- Treatment with PC-mAb(T15) reduced accelerated atherosclerosis by decreasing ER stress markers and CCL2 production - Four human monoclonal PC-mAbs were identified that inhibited macrophage oxLDL-C uptake and diminished vascular inflammation and lesion development	The optimized PC-mAb X19-A05 significantly attenuated vascular inflammation and atherosclerosis in vivo, and the clinical-grade production of ATH3G10 was well tolerated, offering a promising strategy for CVD prevention
Zhong, 2012, [83]	Nasal oxLDL-C vaccine	To explore the effects of nasal administration of oxLDL-C on atherosclerosis and the cellular mechanisms involved in inducing atheroprotective immune responses	- Nasal oxLDL-C administration significantly reduced the initiation (47.6%) and progression (21.1%) of atherosclerosis, with a 35.8% reduction in progression in the enhanced immunization group - These effects were accompanied by increased CD4^+^ LAP+ Tregs and CD4+CD25+FOXP3^+^ Tregs, enhanced TGF-β production, and suppressed Th cell responses - Neutralization of TGF-β partially counteracted the protective effect, indicating that TGF-β is crucial for Tregs to acquire regulatory properties	Nasal oxLDL-C treatment promotes Treg responses to inhibit oxLDL-C-specific T cells and ameliorate atherosclerosis
Klingenberg, 2010, [97]	p210-CTB fusion protein vaccine	To develop a mucosal vaccine targeting an ApoB100 peptide to induce an atheroprotective immune response and reduce atherosclerosis progression	- Nasal administration of the p210-CTB vaccine decreased aortic lesion size by 35% in ApoE(−/−) mice - The vaccine induced regulatory T cells that suppressed effector T cell responses to ApoB100 and increased IL-10-positive CD4^+^ T cells, along with a peptide-specific antibody response	The vaccine provided atheroprotection even in mice with non-functional TGF-β receptors on T cells, demonstrating its potential as an effective strategy for modulating immune responses against LDL-C
Fang, 2024, [122]	PCSK9 NP vaccine	To develop a NP-based vaccine targeting PCSK9 and evaluate its effects on hypercholesterolemia and atherosclerosis in mouse models	- The PCSK9 NP vaccine elicited interfering antibodies against PCSK9, reducing serum lipid levels in both high-fat diet-induced and adeno-associated virus hPCSK9D374Y-induced hypercholesterolemia models - The vaccine also significantly decreased aortic plaque lesions and macrophage infiltration in an atherosclerosis mouse model - The vaccine’s effectiveness depended on Tfh cells and LDLr	The PCSK9 NP vaccine is a promising therapy for hypercholesterolemia and atherosclerosis
Rittershaus, 2000, [132]	CETP peptide vaccine	To evaluate the effects of a peptide vaccine targeting CETP on lipoprotein metabolism and atherosclerosis in cholesterol-fed rabbits	- CETP vaccination reduced plasma CETP activity and altered the lipoprotein profile, increasing HDL-C by 42% and decreasing LDL-C by 24% - The atherosclerotic lesion area in the aorta was reduced by 39.6% in vaccinated rabbits compared to controls	CETP activity inhibition via vaccination has anti-atherogenic effects, highlighting the potential of vaccines targeting self-antigens for therapeutic use in managing atherosclerosis
Fukami, 2021, [135]	ANGPTL3 E3 peptide vaccine	To develop a peptide vaccine targeting ANGPTL3 and evaluate its effects on obesity-associated dyslipidemia and atherosclerosis in mouse models	- Vaccination with the E3 peptide significantly reduced circulating TGs, LDL-C, and sd-LDL-C levels in ob/ob mice while also mitigating obesity-induced fatty liver without inducing cytotoxicity - In ApoE-deficient mice fed a high-cholesterol diet, E3 vaccination attenuated atherosclerosis progression	ANGPTL3-targeted vaccination is a promising therapeutic strategy for dyslipidemia and related diseases
Hu, 2018, [150]	Vaccine against HSP60	To explore the immunological effects of oral and sc administration of HSP60 as a potential vaccine against atherosclerosis in ApoE−/− mice	- Oral administration of HSP60 significantly reduced plaque size at the aortic root and enhanced regulatory immune responses, including increased M-MDSCs in the blood and spleen, elevated IL-10 levels, and the upregulation of anti-inflammatory markers (FOXP3, Arg1, iNOS mRNA) - Sc administration produced opposite effects, promoting pro-inflammatory responses with G-MDSCs predominance	Oral administration is suggested as the optimal method for HSP60 application to prevent and treat atherosclerosis
Tissot et al., 2013, [153]	IL-1α-C-Qβ vaccine	To investigate whether a vaccine inducing neutralizing antibodies against IL-1α can reduce inflammation and atherosclerosis progression in ApoE(−/−) mice	- IL-1α-C-Qβ vaccination reduced plaque progression by 50% in the descending aorta and 37% at the aortic root - Macrophage infiltration decreased by 22%, and peri-aortic inflammation was reduced by 54%, alongside lower expression of VCAM-1 and ICAM-1	IL-1α vaccination effectively reduces vascular inflammation and atherosclerosis progression, presenting a promising therapeutic option for managing atherosclerosis
Zhou et al., 2016, [159]	ATRQβ-001	To assess the efficacy of the ATRQβ-001 vaccine in preventing atherosclerosis in ApoE−/− mice and its effect on the renin–angiotensin system compared to valsartan	- The ATRQβ-001 vaccine significantly mitigated the atherosclerotic lesion area and improved plaque stability - The ATRQβ-001 vaccine decreased macrophage infiltration, the expression of adhesion molecules, and MCP-1 while reducing endothelial apoptosis induced by AngII - The vaccine also lowered levels of lectin-like oxidized LDL-C receptor-1 and AT1R in the aorta without eliciting feedback in the renin–angiotensin system, outperforming valsartan in this regard	The ATRQβ-001 vaccine effectively mitigates atherosclerosis progression with a unique mechanism of action
Ma, 2023, [167]	ATS7vac	To evaluate the efficacy of three potential vaccines derived from ADAMTS-7 epitopic peptides in preventing atherosclerosis and vascular intimal hyperplasia	- ATS7vac effectively inhibited intimal thickening in murine carotid artery ligation models and alleviated neointima formation in murine wire injury models - ATS7vac reduced atherosclerotic lesions in hyperlipidemic ApoE−/− and LDLr−/− mice and mitigated intimal hyperplasia in swine stented coronary arteries without lowering lipid levels or causing significant immune-related organ injuries - Specific antibodies against ADAMTS-7 inhibited ADAMTS-7-mediated degradation of COMP and TSP-1, curbing VSMC migration and promoting re-endothelialization	ATS7vac represents a novel vaccine approach that complements lipid-lowering therapies for atherosclerotic diseases

Abbreviations: ADAMTS-7: A disintegrin and metalloproteinase with thrombospondin motifs 7; AngII: angiotensin II; APOB100: apolipoprotein B100; ApoE(−/−): apolipoprotein E knockout (mice); ARG1: arginase 1; ATS7vac: ADAMTS-7-derived peptide vaccine; CCL2: C-C motif chemokine ligand 2; CETP: cholesteryl ester transfer protein; CETP peptide: cholesteryl ester transfer protein peptide vaccine; COMP: cartilage oligomeric matrix protein; FOXP3: Forkhead box protein P3; G-MDSCs: granulocytic myeloid-derived suppressor cells; HSP60: heat shock protein 60; IL: interleukin; IL-1α-C-Qβ: interleukin-1 alpha conjugated to Qβ virus-like particle; LAP: latency-associated peptide; LDL-C: Low-density lipoprotein cholesterol; LDLr: Low-density lipoprotein receptor; M-MDSCs: monocytic myeloid-derived suppressor cells; MCP-1: monocyte chemoattractant protein-1; NP: nanoparticle; PC: phosphatidylcholine; PC-mAb(T15): chimeric anti-PC monoclonal antibody (T15); PCSK9: proprotein convertase subtilisin/kexin type 9; SC: subcutaneous; sd-LDL-C: small dense low-density lipoprotein cholesterol; TGF-β: transforming growth factor-beta; Tfh cells: T follicular helper cells; Tregs: regulatory T cells; VCAM-1: vascular cell adhesion molecule 1; VSMC: vascular smooth muscle cells.

## Data Availability

Not applicable.

## Abbreviations

ApoB: apolipoprotein B; ApoB100: apolipoprotein B100; APCs: antigen-presenting cells; ApoE: apolipoprotein E; ApoE−/−: apolipoprotein E-deficient (mouse model); ASO: antisense oligonucleotides; ASCVD: atherosclerotic cardiovascular disease; B1 cells: B cell subtype 1; B2 cells: B cell subtype 2; CAD: coronary artery disease; CANTOS: Canakinumab Anti-inflammatory Thrombosis Outcome Study; CAR: chimeric antigen receptor; CCL2: C-C motif chemokine ligand 2; CCL5: chemokine (C-C motif) ligand 5; CCR5: chemokine receptor 5; CD: cluster of differentiation; CD8 mAb: CD8-specific monoclonal antibody; CETP: cholesteryl ester transfer protein; CeVD: cerebrovascular disease; CFA: Complete Freund’s Adjuvant; CI: confidence interval; CKD: chronic kidney disease; COLCOT: Colchicine Cardiovascular Outcomes Trial; CRISPR: clustered regularly interspaced short palindromic repeats; CRP: C-reactive protein; CV: cardiovascular; CVD: cardiovascular disease; CXCL: C-X-C motif chemokine ligand; DC: dendritic cell; ECM: extracellular matrix; EGFA: epidermal growth factor-like repeat A; FDA: Food and Drug Administration; FDG-PET: fluorodeoxyglucose–positron emission tomography; FOXP3: Forkhead box protein P3; GM-CSF: granulocyte–macrophage colony-stimulating factor; HLA: human leukocyte antigen; HR: hazard ratio; hsCRP: high-sensitivity CRP; HSP: heat shock protein; ICAM-1: intercellular adhesion molecule 1; IFN-γ: interferon gamma; IgG: immunoglobulin G; IgM: immunoglobulin M; IL: interleukin; IL-1R1: interleukin-1 receptor type I; ILC: innate lymphoid cell; LDL-C: low-density lipoprotein cholesterol; LDLr: low-density lipoprotein receptor; LILACS: Low-Dose Interleukin-2 in Patients with Stable Ischemic Heart Disease and Acute Coronary Syndromes; LNPs: lipid nanoparticles; LO-chip: lymphoid organ-on-a-chip; Lp(a): lipoprotein(a); LPL: lipoprotein lipase; LPS: lipopolysaccharide; LoDoCo2: Low Dose Colchicine 2; mAb: monoclonal antibody; MACE: major adverse cardiovascular event; M-CSF: macrophage colony-stimulating factor; MDA: malondialdehyde; MDSCs: myeloid-derived suppressor cells; Mfs: macrophages; MHC: major histocompatibility complex; MHC-II: major histocompatibility complex class II; MI: myocardial infarction; MMP-9: matrix metalloproteinase-9; MPS: microphysiological system; mRNA: messenger ribonucleic acid; NF-κB: nuclear factor kappa-light-chain-enhancer of activated B cells; NK: natural killer; NLRP3: NOD-like receptor protein 3; NSAIDs: nonsteroidal anti-inflammatory drugs; OoC: organ-on-a-chip; OxLDL-C: oxidized low-density lipoprotein cholesterol; PAD: peripheral arterial disease; PAM: peptide amphiphile micelle; PBMCs: peripheral blood mononuclear cells; PC: phosphorylcholine; PCSK9: proprotein convertase subtilisin/kexin type 9; Qβ: virus-like particle platform for vaccine development; RAAS: renin–angiotensin–aldosterone system; RESCUE: IL-6 inhibition with ziltivekimab in patients at high atherosclerotic risk; RRR: relative risk reduction; SARS-CoV-2: severe acute respiratory syndrome-coronavirus-2; STEMI: ST-segment elevation myocardial infarction; TF: tissue factor; Tfh: T follicular helper; TGF-β: transforming growth factor-beta; Th: T helper; TIMP-1: tissue inhibitor of metalloproteinase-1; TP: tetanus peptide; Treg: regulatory T cell; Tregs: regulatory T cells; TT: Tetanus toxoid; VCAM-1: vascular cell adhesion molecule 1; VLDL: very low-density lipoprotein; VLP: virus-like particle; VSMC: vascular smooth muscle cell.
